# Comparative genomics of Bacteria commonly identified in the built environment

**DOI:** 10.1186/s12864-018-5389-z

**Published:** 2019-01-28

**Authors:** Nancy Merino, Shu Zhang, Masaru Tomita, Haruo Suzuki

**Affiliations:** 1 Earth-Life Science Institute, Tokyo Institute of Technology, Ookayama, Meguro-ku, Tokyo, 152-8550 Japan; 2 Department of Earth Sciences, University of Southern California, Stauffer Hall of Science, Los Angeles, CA 90089 USA; 3 Global Research Center for Environment and Energy based on Nanomaterials Science, National Institute for Material Science, 1-1 Namiki, Tsukuba, Ibaraki, 305-0044 Japan; 4 Section of Infection and Immunity, Herman Ostrow School of Dentistry of USC, University of Southern California, Los Angeles, CA 90089-0641 USA; 5 Faculty of Environment and Information Studies, Keio University, Fujisawa, Kanagawa 252-0882 Japan; 6 Institute for Advanced Biosciences, Keio University, Tsuruoka, Yamagata, 997-0035 Japan

**Keywords:** Built environment, Bacteria, Diversity, Genomic features, Genome size, GC content, Replication strand skew, Codon usage bias

## Abstract

**Background:**

The microbial community of the built environment (BE) can impact the lives of people and has been studied for a variety of indoor, outdoor, underground, and extreme locations. Thus far, these microorganisms have mainly been investigated by culture-based methods or amplicon sequencing. However, both methods have limitations, complicating multi-study comparisons and limiting the knowledge gained regarding in-situ microbial lifestyles. A greater understanding of BE microorganisms can be achieved through basic information derived from the complete genome. Here, we investigate the level of diversity and genomic features (genome size, GC content, replication strand skew, and codon usage bias) from complete genomes of bacteria commonly identified in the BE, providing a first step towards understanding these bacterial lifestyles.

**Results:**

Here, we selected bacterial genera commonly identified in the BE (or “Common BE genomes”) and compared them against other prokaryotic genera (“Other genomes”). The “Common BE genomes” were identified in various climates and in indoor, outdoor, underground, or extreme built environments. The diversity level of the 16S rRNA varied greatly between genera. The genome size, GC content and GC skew strength of the “Common BE genomes” were statistically larger than those of the “Other genomes” but were not practically significant. In contrast, the strength of selected codon usage bias (S value) was statistically higher with a large effect size in the “Common BE genomes” compared to the “Other genomes.”

**Conclusion:**

Of the four genomic features tested, the S value could play a more important role in understanding the lifestyles of bacteria living in the BE. This parameter could be indicative of bacterial growth rates, gene expression, and other factors, potentially affected by BE growth conditions (e.g., temperature, humidity, and nutrients). However, further experimental evidence, species-level BE studies, and classification by BE location is needed to define the relationship between genomic features and the lifestyles of BE bacteria more robustly.

**Electronic supplementary material:**

The online version of this article (10.1186/s12864-018-5389-z) contains supplementary material, which is available to authorized users.

## Background

The microbial community of the built environment (BE) is an important player in human-microbe interactions. As such, in order to build urban environments that benefit human well-being, it is necessary to study the relationship between the BE and microbial communities. As of 2016, about 54% of the world’s population is living in urban areas [1], and by 2050, this number is expected to increase to 66% [2]. Moreover, people spend about 87% of their time indoors and about 6% in cars [3], suggesting that the indoor microbial community can play an important role in the lives of individuals. In fact, the indoor microbial community has already been shown to affect occupant health (e.g., respiratory health [4] and asthma [5]), including adverse effects on mental health [6], and can be influenced by building design (e.g., ventilation), occupants, and usage [7–9]. In turn, individuals can easily influence the surrounding microbial community with their own personal microbiome, especially through physical contact [10–12] and movement [13], leaving a microbial fingerprint in the built environment [9, 14, 15]. The microbial community of the BE also extends to the outdoor (e.g., green roofs [16] and parks [17]), underground (e.g., transit systems [18–20]), and extreme environments (e.g., cleanrooms [21] and space [21, 22]).

The BE microbiome is slightly influenced by environmental conditions, mainly temperature, humidity, and lighting [23–28]. Several other building parameters have been tested previously (e.g., room pressure, CO_2_ concentration, surface material) but were not found to play a significant role in the microbial community composition [29, 30]. Moisture levels are widely known to affect microbial abundances and activity, especially when water damage occurs (e.g., flooded homes had higher abundances of *Penicillium* [31]). However, many indoor built environments are largely devoid of water and nutrients, and it is likely that geographical location, on the scale of cities or even at larger scales [32], plays a more important role in the microbiome composition [30].

The relationship between humans and microorganisms in the BE has moved from investigations limited to culture-based methods to approaches involving next-generation sequencing. One of the first publications on an indoor microbial community occurred in 1887 [33], which expounded a positive correlation between the presence of indoor microorganisms and death rate. Since the advent of high-throughput sequencing, several studies have used amplicon sequencing to gain more information about the microbial community of the BE, including the ribosomal RNA region (e.g., 16S rRNA) for Bacteria and Archaea and the internal transcribed spacer (ITS) region for Fungi [29]. The microbial communities of a variety of locations have been analyzed, such as clean rooms [21], operating rooms [34], plumbing systems [35], universities [36], and transit systems [18–20]. While these studies have enhanced our understanding of the relationship between humans, microorganisms, and the built environment [25, 29, 37], there are limitations to amplicon sequencing, including bias with sequencing primers, targeted amplicon region, DNA extraction protocols, and sequencing platforms [38], which make multi-study comparisons difficult.

Improving our understanding of microbial communities in the BE can be achieved by analyzing draft or complete genomes derived from genomic and metagenomic studies [39]. There have been several published genomes of bacteria collected from the BE, such as *Dermacoccus nishinomiyaensis* [40], *Arthrobacter* sp. [41], and *Gordonia* sp. [42], among others [43–53]. These data provide detailed information on individual bacterial genomes and can be indicative of a bacteria’s lifestyle or ecological niches [54, 55]. For example, comparative genomics of *Lactobacillus* species, a common microorganism in the human vagina which is mostly absent from other habitats, revealed that the genomes of the vaginal species were smaller with lower GC (guanine and cytosine) content compared to the non-vaginal species [56]. The observed genome size reduction suggests that the vaginal *Lactobacillus* species has “some degree of adaptation to a host-dependent lifestyle” and is commonly observed in symbiotic microorganisms [56]. However, the individual organismal genome information (e.g., genome size and nucleotide composition) has not been investigated in depth for microorganisms in the BE.

In the present study, we performed genome sequence analyses for bacteria that have been commonly identified in BEs, and focused on genomic features, including genome size, GC content, replication strand skew, and codon usage bias. This information could be useful for the characterization of the microbial members present in BEs, and in the future, these basic features might be useful to help predict the microorganisms likely to adapt to BE conditions.

## Results

### Bacteria commonly identified in the built environment

Built environments (BEs) are occupied by various microorganisms and are also important transitions that link the natural world, humans, and the urban environment. The indoor microbiome has already been shown to influence human health [4–6], and a building’s design and operation can play a major role in the spread of microorganisms, including pathogens [25]. For example, air and water via ventilation and plumbing systems, respectively, are major routes for microbial dispersal throughout a BE [25]. Since BEs are designed to improve the lives of the individuals cohabiting them, it is important to understand the relationship between the BEs and the microorganisms therein.

In this study, we selected 28 bacterial genera that have been commonly identified in the BE at the genera level from 54 publications (Additional file 1: Table S1–S2), ranging from various locations around the world (Additional file 2: Figure S1) and covering four major BE locations (indoor, outdoor, underground, and extreme), several sub-locations (e.g. hospital, residential, recreation, space, subway, and cleanroom), climates, and 3 sample types (surface, air, and water) (Table 1, Additional file 1: Table S3-S5). The International Space Station (ISS) is included as a built environment located in space (or low Earth orbit), and the microorganisms observed in this location would be affected by microgravity and increased radiation. The list of common BE bacterial genera (“Common BE genera”) was obtained by selecting genera that have been identified in over 10% of the total publications (*n* ≥ 6 publications) and have at least one completed genome in the NCBI RefSeq database (*n* = 28 genera) (Additional file 1: Table S1). The “Common BE genera” and their identified locations in the BE are summarized in Table 1.

**Table 1:** Locations in the BE where “Common BE genera” were identified. The locations where “Common BE genera” were identified are listed for the 28 genera. This list is based on the 54 publications used for this study (see Additional file 1: Table S2)

Bacterial Genera	Environment Type in BE	Ref
*Acinetobacter*	Clinical (e.g., hospitals), Residential (e.g., bathroom), Extreme (e.g., spacecraft, cleanroom. ISS), Subway (e.g., underground touchscreens). Public recreation (e.g., gym), Hotel bathroom, Office workspace, University (e.g., classroom)	[18, 30, 32, 36, 133–144]
*Arthrobacter*	Extreme (e.g, cleanroom, ISS), Residential dust, Subway air	[21, 22, 141, 145–149]
*Bacillus*	Clinical (e.g., hospitals), Residential (e.g., bathroom), Extreme (e.g., spacecraft, cleanroom, ISS), Subway, Public recreation (e.g., gym), Hotel bathroom, Office workspace	[18, 21, 22, 133, 135, 136, 138, 139, 141–143, 147, 148, 150–156]
*Bradyrhizobium*	Extreme (e.g., spacecraft, cleanroom, ISS), Residential (e.g., wall surfaces), Clinical (e.g., hospital bathroom), Office workspace, Hotel bathroom	[135, 136, 139, 141, 150, 156–159]
*Brevundimonas*	Clinical (e.g., hospital), Extreme (e.g., spacecraft, cleanroom, ISS), Subway, University classroom	[12, 18, 21, 22, 135, 141, 143, 157, 160]
*Burkholderia*	Extreme (e.g., spacecraft, cleanroom, ISS), Residential (e.g., bathroom), Clinical (e.g., hospital), Hotel, bathroom Public recreation (e.g., park, gym)	[112, 136, 137, 141, 146, 149, 155, 161]
*Clostridium*	Residential (e.g., kitchen), Extreme (e.g., cleanroom, ISS), Subway	[20, 21, 138, 141, 154, 156, 162]
*Corynebacterium*	Clinical (e.g., hospitals), Residential (e.g., dust), Extreme (e.g., spacecraft, cleanroom, ISS), Subway (e.g., ticketing machines, underground touchscreens), Office, workspace University (e.g., classroom, dormitory)	[10, 12, 20, 21, 32, 36, 133, 135, 138, 140–145, 150, 153–156, 158, 163–166]
*Delftia*	Extreme (e.g., spacecraft, cleanroom, ISS), Clinical (e.g., hospital)	[21, 135, 137, 139, 141, 146, 156, 157, 160, 163]
*Enterobacter*	Extreme (e.g., spacecraft, ISS), Subway (e.g., outdoor and underground surfaces), University (e.g., classroom,)	[18, 135, 137, 156, 164, 167]
*Enterococcus*	Extreme (e.g., cleanroom, ISS), Clinical (e.g., hospital), Subway (e.g., outdoor and underground surfaces), Public recreation (e.g., park)	[18, 30, 137, 139, 142, 153, 161]
*Escherichia*	Clinical (e.g., hospitals), Residential (e.g., kitchen, bathroom), Extreme (e.g., ISS), Subway (e.g., passenger area), Public recreation (e.g., gym), Hotel bathroom	[136, 137, 153, 157, 160, 162, 168, 169]
*Kocuria*	Residential (e.g., indoor surface), Extreme (e.g., cleanroom, ISS), Subway (e.g., underground air), Clinical (e.g., hospitals)	[21, 22, 112, 138, 142–144, 147, 148, 167]
*Lactobacillus*	Clinical (e.g., nursing home), Residential (e.g., indoor air, surface dust), Extreme (e.g., cleanroom, ISS), Subway (e.g., touchscreens), Office workspace) University (e.g., classroom, dormitory, bathroom)	[10, 12, 13, 21, 30, 32, 36, 141, 144, 145, 150, 156, 165, 166]
*Methylobacterium*	Clinical (e.g., hospitals), Residential (e.g., bathroom), Extreme (e.g., spacecraft, cleanroom, ISS), Subway (e.g., touchscreens), Office (e.g., dust), University (e.g. door handle), Hotel bathroom	[13, 21, 22, 32, 36, 135, 136, 139, 141, 144, 146, 154, 157, 163, 167, 170]
*Microbacterium*	Extreme (e.g., spacecraft, cleanroom, ISS), Subway (e.g., underground air)	[21, 22, 135, 139, 147, 148]
*Micrococcus*	Clinical (e.g., hospitals), Residential (e.g., indoor air, surface), Extreme (e.g., spacecraft, cleanroom, ISS), Subway (e.g., underground air)	[20, 22, 32, 112, 133, 135, 138, 142–144, 147, 148, 153, 155, 167]
*Mycobacterium*	Clinical (e.g., hospitals), Residential (e.g., indoor air, surface), Extreme (e.g., cleanroom), Subway (e.g., outdoor air), Hotel (e.g., showerhead), Public recreation (e.g., gym)	[134, 136, 137, 141, 143, 144, 159, 163, 171–173]
*Neisseria*	Clinical (e.g., hospitals), Residential (e.g., dust), Extreme (e.g., ISS), Hotel (e.g., showerhead), Public recreation (e.g., gym), Office workspace	[136, 145, 150, 154, 156, 158]
*Paenibacillus*	Extreme (e.g., space station, ISS), Subway (e.g., underground air)	[21, 22, 135, 147, 148, 156]
*Prevotella*	Residential (e.g., wall surface, dust), Extreme (e.g., ISS), Office workspace, University (e.g., dormitory)	[21, 32, 145, 150, 158, 165]
*Propionibacterium*	Clinical (e.g., nursing home), Residential (e.g., kitchen, bathroom), Extreme (e.g., cleanroom, space station), Subway (e.g., indoor air), University (e.g., classroom, door handle)	[20, 21, 32, 36, 133, 143, 144, 151, 154, 156, 157, 164–166]
*Pseudomonas*	Clinical (e.g., hospitals), Residential (e.g., kitchen, bathroom), Extreme (e.g., cleanroom, space station, ISS), Subway (e.g., underground air), University (e.g., door handle), Hotel (e.g., showerhead), Public recreation (e.g., gym), Office (workspace)	[18, 21, 22, 30, 36, 112, 134–137, 141–144, 148–151, 153, 156, 163, 172, 174, 175]
*Ralstonia*	Clinical (e.g., hospitals), Residential (e.g., indoor air), Extreme (e.g., cleanroom, space station, ISS)	[135, 139, 141, 146, 149, 152, 157, 167]
*Sphingomonas*	Clinical (e.g., hospitals), Residential (e.g., bathroom), Extreme (e.g., cleanroom, space station, ISS), Subway (e.g., ticketing machines, underground touchscreens), University (e.g., classroom), Hotel (e.g., showerhead), Public recreation (e.g., gym, park, parking lot), Office (e.g., dust)	[13, 21, 22, 32, 36, 134–138, 140, 141, 144, 146, 149, 154, 156, 157, 159, 161, 163, 164, 170]
*Staphylococcus*	Clinical (e.g., hospitals), Residential (e.g., bathroom), Extreme (e.g., cleanroom, space station, ISS), Subway (e.g., air), University (e.g. classroom), Hotel (e.g., showerhead), Public recreation (e.g., gym), Office workspace	[12, 13, 20–22, 30, 32, 36, 112, 133, 135–145, 148, 151–156, 160, 163, 164, 166–168, 171, 173]
*Stenotrophomonas*	Clinical (e.g., hospitals), Extreme (e.g. cleanroom, space station, ISS), Subway (e.g. ticketing machines, underground touchscreens)	[18, 21, 22, 141, 149, 157, 160]
*Streptococcus*	Clinical (e.g., hospitals), Residential (e.g., bathroom, wall surface), Extreme (e.g., cleanroom, ISS), Subway (e.g., indoor air, touchscreens), University (e.g., classroom, door handle), Hotel (e.g., showerhead), Public recreation (e.g., gym), Office (e.g., dust, workspace)	[12, 13, 32, 36, 133, 136, 137, 139, 141, 142, 144, 145, 150, 151, 153, 154, 156, 158, 160, 164, 166, 171, 173]

From the 54 publications used in this study, many of the “Common BE genera” (Table 1) were identified around the world (Additional file 2: Figure S1). For example, *Acinetobacter* was found in five countries, spanning eight different climates, and in the ISS. Unsurprisingly, all 28 genera had some association with humans, as analyzed by MetaMetaDB (Additional file 1: Table S6) [57], further demonstrating the influence that humans have on the BE microbiome [29, 37]. Due to the limitations of this study, the prevalence of these “Common BE genera” cannot yet be associated with BE selection pressures. For example, while there are several other human-associated genera (e.g., *Haemophilus, Veillonella, Alistipes, Rothia*), the microbial community abundances could be affected by different abundance levels and shedding rates across the human body. Other limitations are listed in the section “Robustness and limitations.”

### Diversity among common BE genera

To assess the diversity of the “Common BE genera,” we calculated the mean distance (Dmean) between all pairs of taxa within each genus based on 16S rRNA gene sequences available in the LTP datasets of the SILVA v128 release [58]. The SILVA database was selected over other 16S rRNA databases (e.g. Greengenes [59, 60] and RDP [61]) due to greater alignment quality [62] and because it is continuously updated [63]. The Dmean was also selected over the phylogenetic diversity index (PD) [64, 65] because it is less affected by the number of taxa (N) available in the LTP database, as demonstrated by a smaller Pearson correlation coefficient (*r* = 0.0017) between N and Dmean compared to N and PD (*r* = 0.7248) (Additional file 2: Figure S2).

**Fig. 1:**
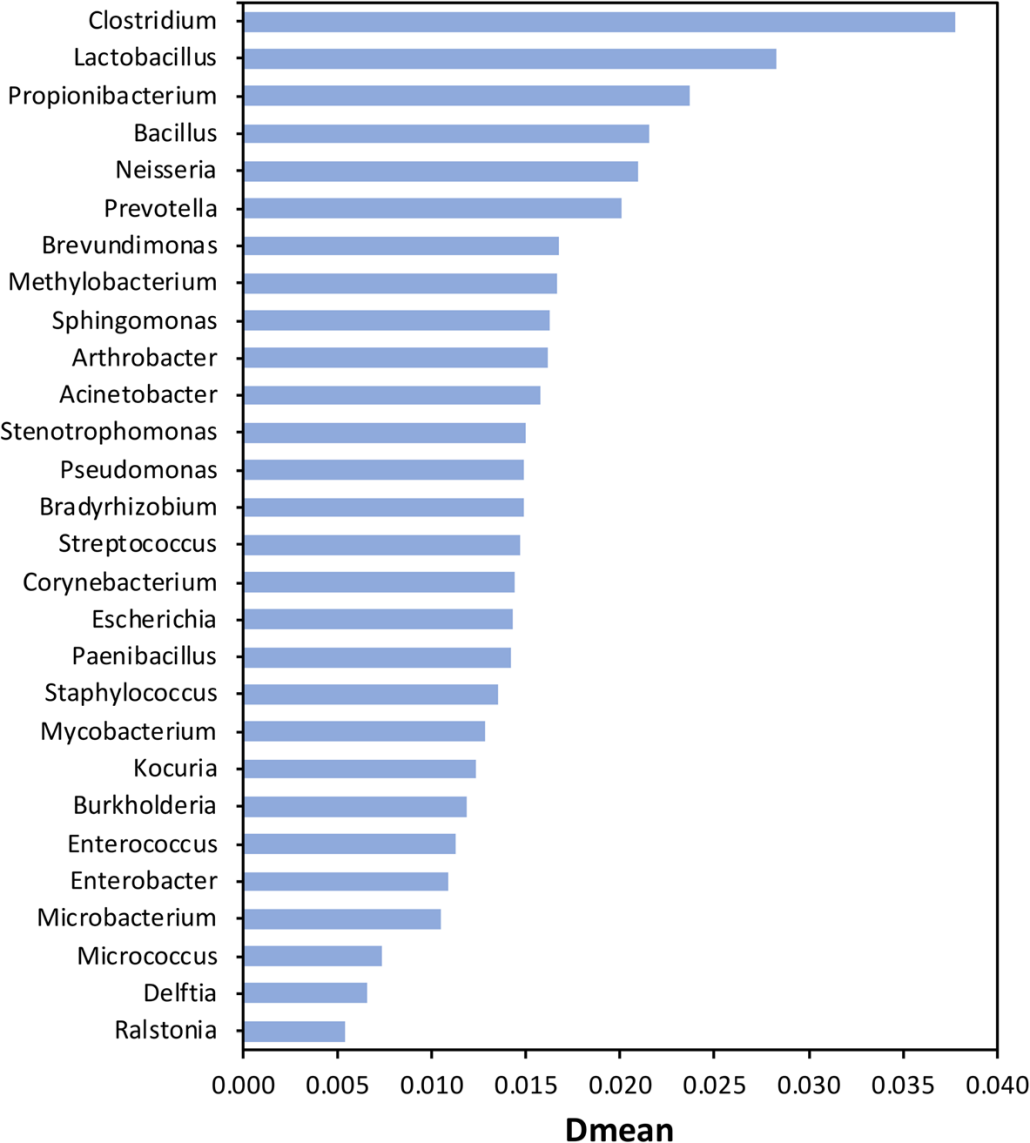
Diversity levels in 16S rRNA gene sequences for each bacterial genus commonly found in the built environment. The mean distance (Dmean) between all pairs of bacteria was used as a diversity index [58]

The Dmean for each “Common BE genus,” with *n* > 2 in the LTP database ranged from 0.005 (*Ralstonia*) to 0.038 (*Clostridium*) with a median value of 0.015 (Fig. 1, Additional file 1: Table S7), suggesting, for example, that taxa within *Ralstonia* are relatively more closely related than those in *Clostridium*. In comparison, the Dmean for genera not commonly found in the BE (850 genera) ranged from 0 (*Stigmatella*) to 0.115 (*Salinibacter*) with a median value of 0.016. A Wilcoxon rank sum test, which compared the Dmean values between the two groups (28 genera versus 850 genera), was not statistically significant (*p*-value = 0.28). This indicates that there was insufficient evidence to conclude that there was a significant difference in intra-genus diversity between “Common BE genera” and “Other genera.” However, the 16S rRNA gene has its limitations (e.g., sequence heterogeneity [66] and horizontal gene transfer [67]), even though it is widely used as a molecular clock to understand evolution [67–70]. Intragenus variations in genomic features (genome size, GC content, GC skew, and codon usage bias) can reflect the level of diversity among taxa within each of the “Common BE genus.”

### Genome size, GC content, and GC skew

We compared the genomic features (genome size, GC content, GC skew, and codon usage bias) of 2580 complete prokaryotic genomes from the NCBI RefSeq database, in which 717 genomes are from bacteria commonly identified in the BE (“Common BE genera”) and 1863 other genomes (“Other genera”) (Additional file 1: Table S8-S9). The “Other genomes” have not been identified in at least six publications (equivalent to 10% of the publications used for this study).

Genomic features, including genome size, GC content, and GC skew, can provide information about the bacterial lifestyle as well as phylogeny [54]. For example, genome size can reflect genome streamlining, symbiosis, or genome expansion [71, 72]. GC content has been shown to relate to both the phylogeny and ecological adaptations of a microbial species, as demonstrated by Reichenberger and co-workers [73]. GC content can range from 15 to 75% and can be influenced by environmental factors such as temperature [74], oxygen levels [75], and nucleotide availability [76]. Furthermore, GC skew, as quantified by the GC skew index (GCSI), measures the strength of replication strand skew [77] and could indicate variation in mutational and selective pressures between leading and lagging strands of DNA replication [78]. Indeed, the leading strand tends to be biased with G and T while the lagging strand is rich in A and C [79]. Strand composition bias has been shown to especially occur in obligate intracellular microorganisms that permanently live within a host, resulting in the loss of some DNA repair genes and the accumulation of mutations [80]. Replication, repair, and transcription enzymes are thought to influence strand composition, where different genes are involved in transcribing the leading and lagging strand [81]. Each enzyme will have different mutational and selective pressures, and thus, GCSI informs DNA repair capabilities and provides insight into the metabolism and lifestyle of bacteria [81].

**Fig. 2:**
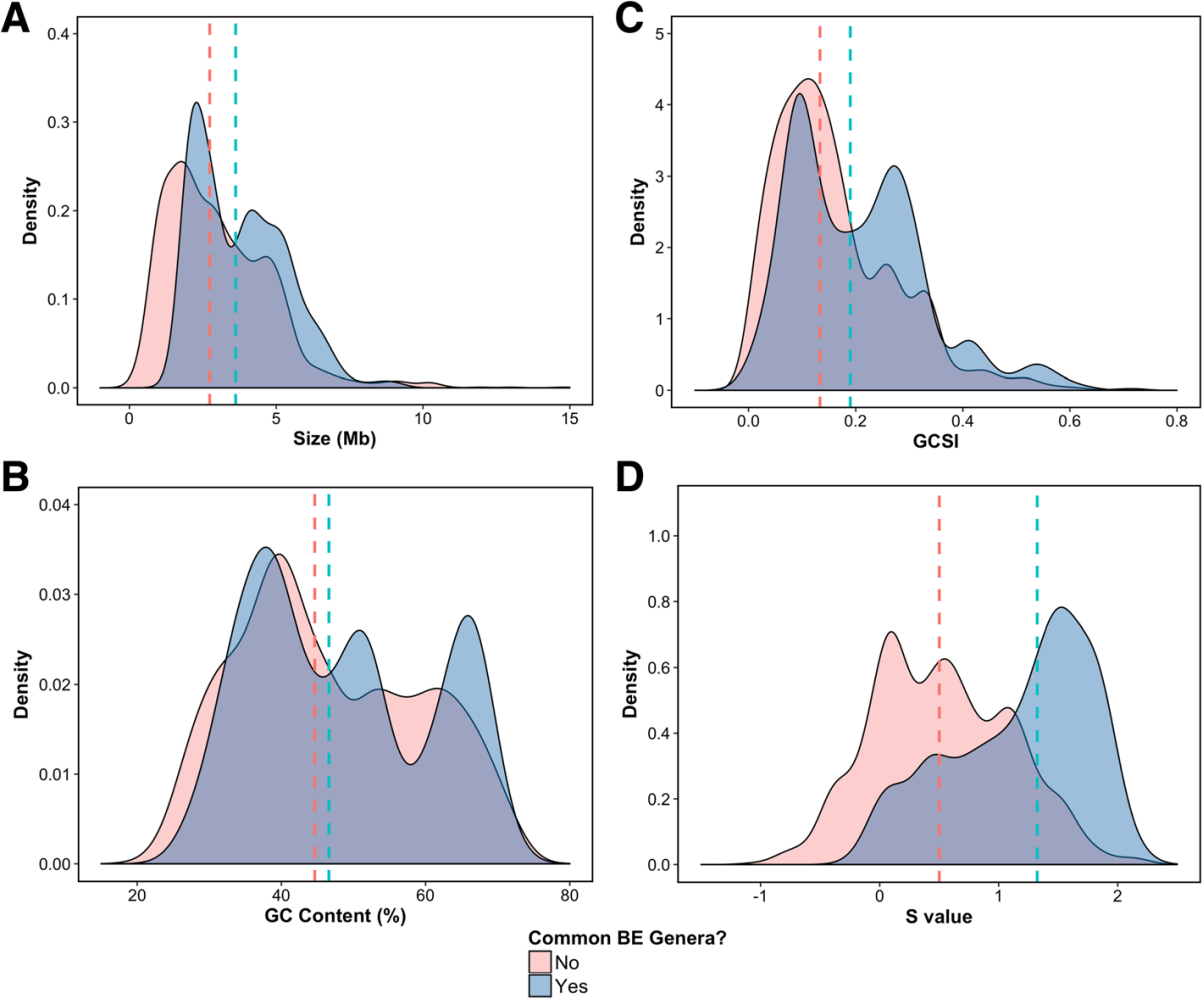
Density plots comparing “Common BE genomes” against “Other genomes.” **a** Genome size, **b** GC Content (%), **c** GCSI, and **d** S value. The dashed lines indicate the median value for “Common BE genomes” (blue) and “Other genomes” (pink). The S value is the only significant genomic feature when comparing “Common BE genomes” against “Other genomes”

**Fig. 3:**
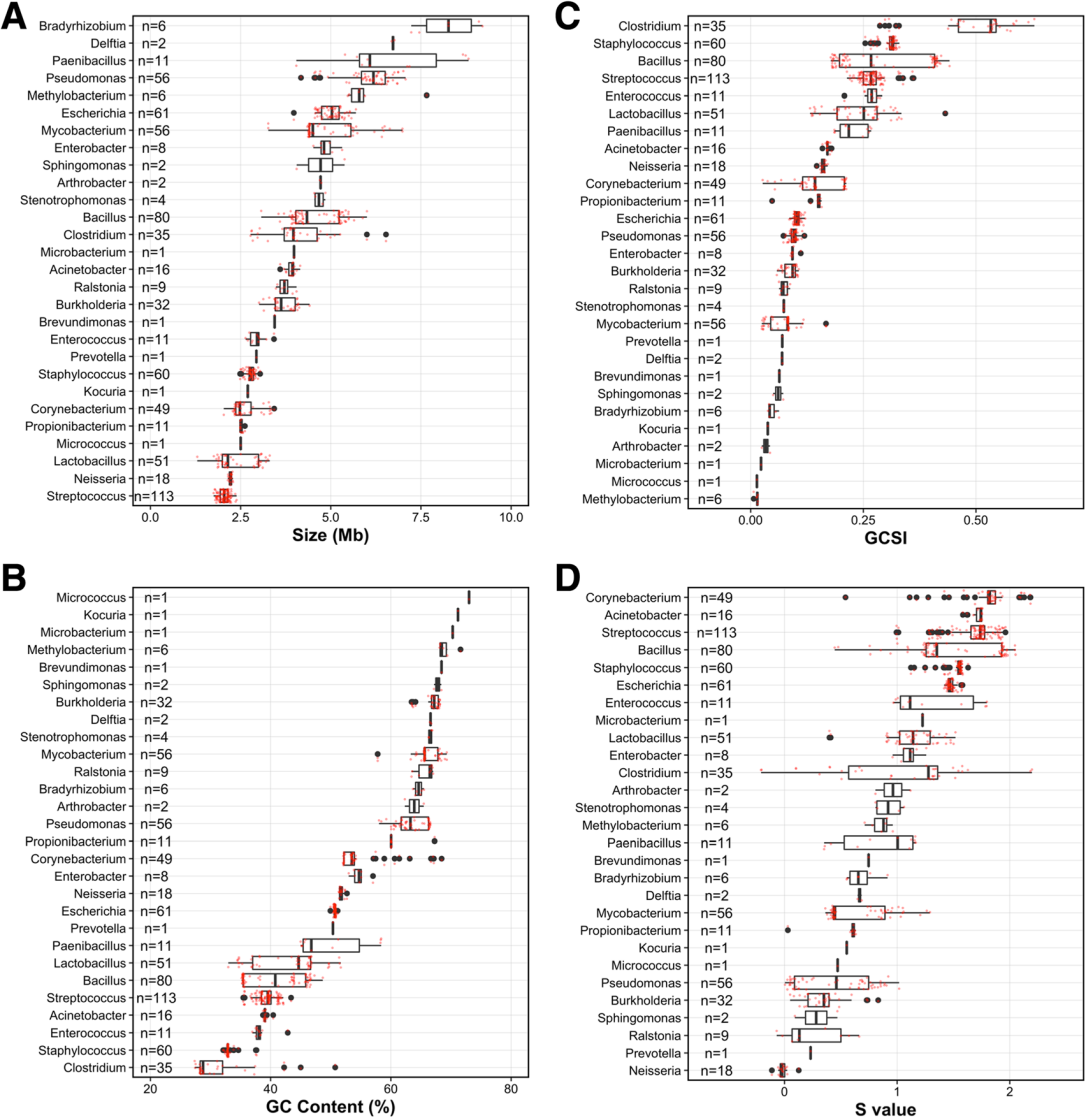
Distribution of each genome feature for the “Common BE genera.” Box-and-whisker plots displaying the distributions of genomic features for each “Common BE genus” based on a five number summary (minimum, 25th percentile, median, 75th percentile, and maximum). Outliers are plotted as black filled circles. Red circles denote individual genomes. Genomic features are as follows: **a** Genome size, **b** GC Content (%), **c** GCSI, and **d** S value

The “Common BE genomes” tended to have larger genome sizes (1.30–9.21 Mb, median 3.62 Mb) (Fig. 2a), higher GC contents (27.4–73.0%, median 46.6%) (Fig. 2b), and higher GCSI (0.007–0.629, median 0.19) (Fig. 2c) compared to the “Other genomes”. Among the 717 “Common BE genomes,” the bacterium, *Clostridium perfringens* strain 13 (NC_003366), had the highest GCSI value (0.629) and exhibited a clear GC skew, especially around 1.4 Mb (Additional file 2: Figure S3A), while *Methylobacterium* sp. 4–46 (NC_010511) had the lowest GCSI value (0.007) with indiscernible GC skew (Additional file 2: Figure S3B). The median for all three features of the “Common BE genomes” was higher than that of the “Other genomes” (1863 genomes; Size = 2.74 Mb; GC content = 44.6%; GCSI = 0.133) (Fig. 2). While these differences were statistically significant based on the Wilcoxon rank sum test q-value (genome size = 1.68e-31; GC content = 0.002; GCSI = 6.46e-17), further analysis using the Cliff’s delta effect size (genome size = 0.3, GC content = 0.079, GCSI = 0.215) demonstrated negligible (< 0.147) or small (< 0.33) thresholds when comparing the “Common BE” and “Other” genomes. Similar results were observed when categorizing by environments (MetaMetaDB) (Additional file 2: Figure S4–S6). Moreover, each genome feature may cover a wide range (Fig. 3a-c), depending on the BE genus.

### Codon usage bias

The genetic code of each “Common BE genus” can also provide information about codon usage bias, which has further implications on evolutionary processes, such as selection, mutation [82], and even horizontal gene transfer [83–85]. Many amino acids can be encoded by more than one codon, also known as synonymous codons, due to the redundancy of the genetic code, and there is generally a preference for one synonymous codon over another [86]. The pattern of synonymous codon usage can vary between organisms (e.g., some organisms use a set of synonymous codons more frequently) and across genes within a genome [82, 87]. It is hypothesized that codons are selected based on their impact on translation, influencing bacterial growth [88, 89], and that codon usage bias can be derived from highly expressed genes [90, 91]. Several studies have demonstrated that codon usage bias correlates with bacterial growth rates, likely suggesting a selection towards efficient translation machinery [87, 89, 92, 93]. Codons may also be selected to optimize protein production speed [94]. For example, the codon usage bias of *Salmonella enterica* serovar Typhimurium, a fast-growing bacterium, correlates well with gene expression levels [87]. Thus, it is imperative to determine the codon usage bias in order to further surmise the lifestyles of bacteria that have been commonly identified in the BE.

Here, we determined the strength of selected codon usage bias (S value) (Fig. 2d), as discussed by Sharp and co-workers [87]. The S value is based on a comparison of codon usage between constitutively highly expressed genes and the entire genome (see Methods for details) [87]. The median S value of the “Common BE genomes” (1.32) was higher than that of the “Other genomes” (0.50), with a large effect size (Cliff’s delta of 0.574). Moreover, the Wilcoxon rank sum test provided a significant result with a q-value of 1.22e-111, suggesting that the S value could be more indicative of the type of bacteria commonly observed in the BE compared to other genomic features described previously (genome size, GC content, and GC skew).

Further categorization of the environments (MetaMetaDB) indicates that the S value is stronger for the “Common BE genomes” observed with the human microbiome, as compared to the other “Common BE genomes” (Additional file 1: Table S10 and Additional file 2: Figure S7). Among the 517 “Common BE genomes” for which species were categorized according to environments in MetaMetaDB, the S value tended to be lower in compost-associated “Common BE genomes” than in the other “Common BE genomes” (Cliff’s delta = − 0.647; q-value = 1.01e-21). In contrast, the median S value for the “Common BE genomes” also associated with the category “human” by MetaMetaDB (*n* = 454; median S value = 1.45) was higher than that for the other “Common BE genomes” (*n* = 63; median S value = 0.71). The difference was large based on the effect size (Cliff’s delta = 0.516) and was statistically significant based on the Wilcoxon rank sum test (q-value = 2.53e-10). This trend is also true when examining only the top bacterial genera found in the human microbiome (list taken from Lloyd-Price J, Mahurkar A*,* et al. [95]). The top human microbiome genera that are also commonly found in the BE (*n* = 301 genomes; median S value = 1.50) had significantly higher S values compared to those not commonly found in the BE (*n* = 28 genomes; median S value = 1.08) with a medium effect size (Cliff’s delta of 0.451) and a q-value of 0.0009. This suggests that the human and BE microbiome are interconnected, with bacterial genera trending towards larger S values. However, the limitations of this study (see section “Robustness and limitations”) cannot associate the “Common BE genera” with BE selection pressures.

When examining each “Common BE genus,” the S value was found to cover a wide range (e.g., *Enterococcus*, *Mycobacterium*, and *Bacillus*) (Fig. 3d). Future reports of BE microbial communities could help to resolve the importance of the S value by accurately identifying taxa to the species level and by unifying metadata collection and method protocols. Indeed, the S value has been shown to vary across species, especially for those that are not closely related [96]; e.g., *Clostridium* has the largest S value range (Fig. 3d) and also has the largest Dmean (0.038) (Fig. 1).

### Case study: *Mycobacterium*

As a case study for one of the “Common BE genera”, we further discuss *Mycobacterium* and describe how the four genomic features can be used to surmise the potential lifestyle of bacteria. *Mycobacterium*, a genus with well-known pathogenic species (e.g., *Mycobacterium tuberculosis* and *Mycobacterium bovis*), has one of the largest genome size ranges from 3.3 Mb [*Mycobacterium leprae* Br4923 (NC_011896)] to 7.0 Mb [*Mycobacterium smegmatis* strain MC2 155 (NC_008596)] with a median of 4.5 Mb (Fig. 3a). *Mycobacterium* has been found in several locations, including hospitals, therapy pools, showerheads, water-damaged homes, and cleanrooms (Table 1). One of the major factors determining the presence of *Mycobacterium* in water-damaged homes may be due to transmission from human and pet occupants [32]. The GC content in *Mycobacterium* was relatively high (57.8–69.3%) compared to other “Common BE genera” (27.4–73.0%) (Fig. 3b), where the outlier group (57.8%) was the species *M. leprae* (Additional file 1: Table S8). The smaller genome size and lower GC content of *M. leprae*, an obligate pathogen, are a result of genome reduction which has been well documented [97]. The GCSI ranged from 0.025 [*M. avium* subsp. *paratuberculosis* K-10 (NC_002944); Additional file 2: Figure S8A] to 0.167 [*M. leprae* Br4923 (NC_011896); Additional file 2: Figure S8B]. The S value for *Mycobacterium* ranged from 0.36–1.30, suggesting that either the growth rate of different *Mycobacterium* species present in the BE varies drastically or that some *Mycobacterium* species have more “volatile” codons, as discussed below. For example, *M. tuberculosis* and *M. leprae* have S values in the lower range (0.36–0.45) and also have slow generation times of ~ 1 and 14 d, respectively [87, 98, 99]. In comparison, one of the highest S values (1.3) corresponded to *M. abscessus*, which has a generation time of 4–5 h [100].

## Discussion

### Genomic features relation to the potential lifestyle of bacteria commonly identified in the built environment

To further understand the 28 “Common BE genera,” we analyzed four genomic features: genome size, GC content, GC skew, and codon bias. While our study based itself on the results of previous studies to retrieve the “Common BE genera,” we aimed to demonstrate the potential of using genomic features to provide insight into microbial lifestyles and to describe the trends found in the “Common BE genera” [54]. The “Common BE genomes” tended to have larger genome sizes, higher GC contents, higher GCSI, and larger S values compared to the “Other genomes.” While the differences for all the genomic features were statistically significant based on the Wilcoxon rank sum test, further analysis by the Cliff’s delta effect size demonstrated that the S value is likely a more important genomic feature for bacteria commonly identified in the BE compared to the “Others” analyzed in this study.

This initial analysis could help begin to surmise certain lifestyles of the bacteria commonly found in the BE. For example, the S value has implications on the growth rates of bacteria [89] found in the BE, which may be higher than those found in other environments, and could also be related to higher levels of gene expression [90, 91]. A stronger preference for codon usage bias in the “Common BE genera” may have resulted from a of long-term relationship with humans (e.g., genome reduction in bacteria was associated with the “Neolithic revolution” [101] and “Common BE genera” were found on nineteenth century documents [102, 103]) but further analysis is needed.

Moreover, the preference for certain codons may be related to either directional mutation or specific selection [104]. In the case of directional mutation, it is hypothesized that some codons are more prone to mutation, resulting in lower S values [87]. For example, *Mycobacterium tuberculosis*, one of the “Common BE genera” and pathogen with S values (0.41–0.45) below the “Common BE” and “Other” genome medians (Fig. 3d), has more “volatile” codons relating to antigens, surface proteins, or antibodies which are likely to mutate more than other codons [105]. These help *M. tuberculosis* prevent host-immune system interactions [105]. As for specific selection, it is thought to lead to efficient translation processes and accurate protein synthesis due to the use of more frequent codons by highly expressed genes [104]. This can be a reflection of an organism’s adaptation to an environment, and it is likely that the “Common BE genomes” share “synchronized regulation mechanisms of translational optimization” [106]. Indeed, this has been shown for 11 distinct metagenomes from various environments [106], where, for example, microorganisms living with an abundant food source (whale fall carcass) have translationally optimized genes for energy production and conversion.

The trend towards larger S values in the “Common BE genera” also suggests that these genera can inhabit a wide range of environments [107]. The “Common BE genera” must also contend with chemicals derived from the daily use of personal care and household products (e.g., avobenzone from sunscreen, laureth sulfate from shampoo, and amlodipine from medication used to treat high blood pressure), in addition to human-derived chemicals (e.g., acyl glycerols, which make up the membrane of human cells) [108–110]. For example, *Propionibacterium* has been shown to metabolize triglyceride triolein, a human acylated glycerol, and was found to be co-localized with acylated glycerols on the human body [108]. Since these chemicals can be found in the BE and may be associated with an occupant’s chemical signature [109], future studies are needed to determine how these chemicals may affect the BE microbial community composition (e.g., rural vs. urban environments, change in a product’s formula, etc.).

While not as important as the S value in this study, larger genome sizes could be attributed to the incorporation of regulatory and secondary metabolic genes [72], which may be important for survival in the BE (e.g., aromatics degradation and regulation to environmental stresses). Indeed, the top three major functional pathways annotated for the microbial community found in ambulances were 1) biosynthesis of cofactors, prosthetic groups, and electron carriers, 2) secondary metabolites biosynthesis, and 3) aromatics compound degradation [111].

### Robustness and limitations

This study demonstrates the potential of using the four genomic features (genome size, GC content, GCSI, and S value) to surmise the lifestyle of bacteria. The “Common BE genera” selected in this study have only been commonly identified by culture-based and amplicon-based sequencing studies, which have limitations as described in the Introduction. Although the “Common BE genera” have been detected in multiple BE studies (≥ 6), these bacteria may not be active in the BE. Moreover, although this study is based on completed genomes from the NCBI RefSeq database, the genomes could have been derived from environments not related to the BE. Thus, the conclusions derived from this study serve as a hypothesis for the potential lifestyles of commonly identified BE bacterial genera. Further studies are needed to accurately determine the typical BE genera and the association of BE genera with BE selection pressures.

It is important to note that the results remained similar when different data sets were compared (Additional file 1: Table S9). We tested the robustness to the composition of the genome data set by testing different subsets of bacteria (e.g., phyla of *Proteobacteria*, *Firmicutes*, and *Actinobacteria*), and also by randomly selecting one representative for species that have multiple strains sequenced. Of the four genomic features (genome size, GC content, GCSI, and S value), only the S value showed consistent results and tended to be higher in the “Common BE genera” compared to the “Others.” This indicates that the selected codon usage bias tends to be stronger in the “Common BE genera” than in the “Other genera,” regardless of the datasets used, and that our results were less affected by biases in the available sequenced genomes. We also tested different numbers of publications (*n* = 1, 2, 3, 4, 5, and 6) to select for BE genera. The corresponding numbers of the selected “Common BE genomes” were 1208, 1029, 922, 825, 739, and 717. Even when genera observed in at least 1 out of 54 publications were defined as the “Common BE genera,” the median S value for the “Common BE genomes” (1.14) was higher than that for the “Other genomes” (0.35) with a large effect size (Cliff’s delta of 0.548), and the Wilcoxon rank sum test returning significant result with q-value of 2.59e-126. This is consistent with the results obtained by larger numbers of publications (*n* > 1) to define the “Common BE genera.” Thus, selected codon usage bias tends to be larger in the “Common BE genomes” than in the “Other genomes,” regardless of the genome data set used and criteria to define BE genera.

Our selection of the 28 common bacterial genera is likely biased towards the genera found in certain locations (e.g. fewer publications sampling outdoors and subways compared to indoors and extreme; more publications sampling locations with mild temperate climates) (Additional file 1: Table S3–S5) and sampling type (e.g., fewer publications conducted microbial community analysis of water samples compared to surface and air samples) (Additional file 1: Table S3). In addition, 16S rRNA amplicon sequencing was the dominant method used to determine the microbial community amongst the 54 publications used in this study. Some publications also conducted culture-based studies (e.g. study on airborne bacteria in Tokyo [112]). This introduces bias from the range of protocols used across publications, including sample collection methods (e.g. swab, wipe, air, and storage method), DNA extraction methods, primers used, 16S rRNA target region (e.g. V3–V4, V4, V6–V8), and sequencing methods [113–115]. With advances in sequencing for 16S rRNA (e.g., full-length [116]), genomes, and metagenomes (e.g., longer contigs, accurate base calling) and increased global research collaboration (e.g., MetaSUB [117]), more specific classification of BE microorganisms can be obtained at the species level, allowing for more accurate descriptions in future studies.

After obtaining the 28 “Common BE genera,” we then used the NCBI RefSeq database to obtain completed genomes. Another level of bias arises from using sequenced genomes from the public database (e.g., towards medically and industrially important microorganisms), although there are ongoing “efforts to expand the bacterial and archaeal reference genomes…to maximize sequence coverage of phylogenetic space” [118]. However, this study aimed to demonstrate the capability of using genomic features to characterize the “Common BE genera,” providing a first step towards understanding the potential lifestyles of these bacteria. As more genomes from the BE microbial community are sequenced (e.g., efforts by the MetaSUB International Consortium [117]), much more accurate analyses can be carried out to appropriately examine the microbial lifestyles based on genomic features and functional annotation.

## Conclusions

Twenty-eight bacterial genera were selected to represent the bacteria commonly identified in the BE. Although geographical location, temperature, and humidity are important factors in shaping the BE microbial composition, many of the “Common BE genera” were identified around the world. All the genera have also been observed in the human microbiome. Here, we used genomic features to demonstrate the potential of understanding the lifestyle of bacteria from the genome. Together, the genome size, GC content, and GC skew for the “Common BE genomes” showed trends similar to (were not strongly deviated from) those for the entire data set of completed prokaryotic genomes analyzed obtained from the NCBI database. On the other hand, the strength of selected codon usage bias (S value) for the “Common BE genomes” tended to be significantly higher than that of the “Other genomes.” As such, the S value could be indicative of bacterial growth rates, gene expression, and other evolutionary processes that may play a role in the bacteria present in the BE. Further insights could be gained through more BE studies analyzing locations with fewer publications (e.g., rural, tropical climates, and outdoor), identifying microbial communities at the species-level, and by minimizing cross-study biases.

## Methods

### Selection of common BE bacterial genera, metadata, and genome sequence data

Bacteria commonly identified in the BE are listed in Additional file 1: Table S1 and Table 1. Since most currently available BE studies conducted 16S rRNA amplicon sequencing, the identification was largely limited to the genus level. In this study, 54 total publications (published between 2003 and 2017) were compiled with metadata, including the bacterial genera, BE location identified, sample type, temperature (°C), humidity (%), and approximate climate (Additional file 1: Table S2). These publications either conducted 16S rRNA amplicon sequencing or isolated bacteria from the BE. If the temperature or humidity was not described by the publication, the average over a certain period of time (either the timeframe stated in the publication or the publication year) was obtained from online sources (see Additional file 1: Table S2 for references and timeframe). In order to obtain climate level assignment, the Köppen climate classification scheme was implemented (1981–2010) by determining the closest latitude and longitude to a publication’s described study location [119] (Additional file 1: Table S4). In order to identify the “Common BE genera,” we selected for bacterial genera which were identified in more than about 10% of the publications (*n* ≥ 6 publications) and had at least one genome sequenced in the National Center for Biotechnology Information (NCBI; https://www.ncbi.nlm.nih.gov) RefSeq database [120, 121] (Additional file 1: Table S8) (*n* = 28 genera). These were denoted as “Common BE genomes” or “Common BE genera” while the bacterial genera not selected were denoted as “Other genomes” or “Other genera.” Based on this criterion, 28 genera were retained (Additional file 1: Table S1).

To further understand the potential associated environments of each BE genus, we used MetaMetaDB (data by November 6, 2014 at http://mmdb.aori.u-tokyo.ac.jp) (Additional file 1: Table S6) [57]. MetaMetaDB is a database to search for the possible habitats a microorganism could live in and was made by collecting 16S rRNA sequences. Hits for environmental categories for each common BE genus was based on an identity threshold of 97%, corresponding to the species taxonomic level. Environmental categories on MetaMetaDB are based on the classification used by the NCBI taxonomy, which include categories such as aquatic, soil, human, compost, and more. While these categories are not well-defined and controlled (e.g., there are several categories for human, including human, human gut, human oral, human skin, and others), we used MetaMetaDB to gain insight into the associated environments of each BE genus.

RefSeq chromosome sequence accessions with the NC_ prefix were obtained from the NCBI prokaryotic genome list (ftp://ftp.ncbi.nih.gov/genomes/GENOME_REPORTS/prokaryotes.txt), and complete sequences of prokaryotic chromosomes (GenBank format [122]) were downloaded with the RefSeq accessions using E-utilities on 2018-01-27. In cases where the organism has multiple replicons (chromosomes and plasmids), only the largest chromosome was used for the analysis as a representative replicon of the organism. The final data set included 2580 prokaryotic genomes (142 Archaea and 2438 Bacteria), including 717 genomes of bacteria belonging to the 28 genera commonly found in the BE (“Common BE genomes”) and 1863 other prokaryotic genomes (“Other genomes”). The 717 “Common BE genomes” belonged to 4 phyla: *Firmicutes* (370), *Proteobacteria* (222), *Actinobacteria* (123), and *Bacteroidetes* (2). The 1863 “Other genomes” belonged to 644 genera from 36 phyla, including *Proteobacteria* (875), *Firmicutes* (192), *Actinobacteria* (115), and *Chlamydiae* (110). The “Common BE genomes” and “Other genomes” were linked to the 18 environmental categories in MetaMetaDB: Aquatic, Biofilm, Compost, Food, Freshwater, Hot_springs, Human, Human_gut, Human_lung, Human_nasal_pharyngeal, Human_oral, Human_skin, Marine, Rhizosphere, Rock, Root, Sediment, and Soil. Complete listings of the genomes used in this study, along with the genomic features, are shown in Additional file 1: Table S8.

### Bacterial diversity

To measure the genetic diversity among taxa within a genus, the mean distance (Dmean) between all pairs of bacteria was calculated [58]. The genetic distance between a pair of bacteria was calculated with the K80 model using the ‘dist.dna’ function of the ‘ape’ package of R (https://cran.r-project.org/web/packages/ape) [123]. We used a nucleotide sequence alignment of the 16S rRNA genes in ‘The All-Species Living Tree’ Project (https://www.arb-silva.de/projects/living-tree/) [124]. LTP datasets based on SILVA release 128 were downloaded from the Download page [125].

### Genomic features

#### Genome size

The total number of nucleotides (A + T + G + C) was calculated from the whole nucleotide sequence of each chromosome.

#### GC content (%)

The relative frequency (percentage) of guanine and cytosine (G + C)/(A + T + G + C) was calculated from the whole nucleotide sequence of each chromosome.

#### GC skew index (GCSI)

The asymmetry in nucleotide composition between leading and lagging strands of DNA replication is represented by GC skew (C-G)/(C + G). The strength of GC skew was measured by the GC skew index or GCSI [126] with a window number of 4096. This fixed window number was used to prevent any effects from biased nucleotide composition in coding regions and is based on an average gene length of 1 kb and a genome size of 2–4 Mb [126]. The GCSI values can range from 0 (no GC skew) to approximately 1 (strong GC skew).

#### Strength of selected codon usage bias (S value)

As a measure of translationally selected codon usage bias, the S value was calculated for each chromosome, as described in Sharp and co-workers [87] and Vieira-Silva and Rocha [89], using the codon usage for four amino acids, Phe (TTC and TTT), Tyr (TAC and TAT), Ile (ATC and ATT), and Asn (AAC and AAT). The two codons are recognized by the same tRNA species, and the C-ending codon is recognized more efficiently than T-ending codon. The S value is based on a comparison of codon usage within these synonymous groups between constitutively highly expressed genes (those encoding ribosomal proteins and translation elongation factors) and the entire genome [87, 89].

### Statistical analyses

We performed several statistical analyses to compare the values of the genomic features (genome size, GC content, GCSI, and S value) between two groups of genomes: e.g., “Common BE genomes” versus “Other genomes”; and MetaMetaDB environment-associated “Common BE genomes” (e.g., “Human”) versus other “Common BE genomes” (e.g., not associated with “Human”).

#### Wilcoxon rank sum test

We performed the Wilcoxon rank sum test (also called Mann-Whitney U test) as a non-parametric statistical hypothesis test to compare the values between two groups [127]. The *p*-value obtained by the statistical test was adjusted for multiple comparisons by controlling for the false discovery rate (FDR) [128]. An FDR adjusted p-value (q-value) of 0.05 was used as a threshold for statistical significance.

#### Cliff’s delta effect size

We calculated Cliff’s delta statistic as a non-parametric effect size to estimate the degree of overlap between two distributions [129]. A Cliff’s delta of 0.0 indicates the group distributions overlap completely, whereas a 1.0 or − 1.0 indicates the absence of overlap between the two groups. A positive Cliff’s delta close to 1.0 indicates that the genomic feature values tended to be higher in the “Common BE genomes” than in the “Other genomes.” A negative Cliff’s delta close to − 1.0 indicates that the genomic feature values tend to be lower in the “Common BE genomes” than in the “Other genomes.” Three thresholds were used to determine the magnitude: |d| < 0.147 “negligible,” |d| < 0.33 “small,” and |d| < 0.474 “medium” or “large” [130]. These thresholds are used for two normal distributions [136], equivalent to the original thresholds used by Cliff (1993) [135] to scale the effect size indices to observable phenomena.

### Software

Genome sequence analyses (e.g., calculating genome size, GC content, GCSI, and S value) were performed using the G-language Genome Analysis Environment version 1.9.1 (http://www.g-language.org) [131]. Statistical computing and graph drawing were conducted with R version 3.3.3 (https://www.R-project.org/) [132].

## Additional files

Additional file 1: **Table S1.** Selection of bacterial genera commonly identified in the built environment. Bacterial genera identified in 54 publications were compiled (see Table S2) and commonly identified genera were selected. All bacterial genera identified in more than about 10% of the publications (*n* ≥ 6 publications) with at least one complete reference genome on the NCBI RefSeq database were used in this study (*n* = 28 genera). **Table S2.** Metadata for each reference. 54 publications were compiled, including metadata for location, sub-locations, bacterial genera identified, sample type, climate (Table S4 and S5), temperature (°C), and humidity (%). If temperature or humidity was not described by the publication, the average over a certain period of time (either the timeframe stated in the publication or the publication year) was obtained from online sources. **Table S3.** Publication count for each “Common BE Bacterial Genus” by macro-Level BE location. Macro-level BE Locations included indoor, outdoor, underground, and extreme. Further division by type of sample is also depicted, including surface (S), air (A), water (W). Darker orange color indicates more references identified the genera in the macro BE location and sample type while lighter orange color indicates fewer references. The total number of references for each location and genera are also shown. **Table S4.** Köppen climate classification. Köppen climate classification was used to identify the climate for each publication’s study location. Only the climate assignment between 1981 and 2010 was used for this study. Abbreviation descriptions, latitude, and longitude values are listed. **Table S5.** Publication count for each “Common BE Bacterial Genus” by climate. The climate was identified for each publication’s study location based on the closest Köppen latitude and longitude values and correlated with the Köppen ID (see Table S4 for Köppen assignment). For publications describing general locations (e.g., only provided a U.S. state name), a central location in the region was chosen for latitude and longitude. Publications without location specifics were not included, and publications in space were separated out to “Space” category. Darker orange color indicates more references identified the genera in the macro BE location and sample type while lighter orange color indicates fewer references. The total number of references for each location and genera are also shown. **Table S6.** MetaMetaDB environmental category assignment for each “Common BE Bacterial Genus.” MetaMetaDB is a database to search for the possible habitats a microorganism could live in and was made by collecting 16S rRNA sequences. Environmental categories for each “Common BE bacterial genus” were based on the identity threshold of 97%, corresponding to the species taxonomic level. Every species for each “Common BE genus” is listed with the corresponding environmental category, where “Y” indicates that the species has been previously identified in the category and “N” indicates the species has not been identified in the category. “Hits” indicates the number of 16S rRNA sequences used by the database. **Table S7.** Mean distance (Dmean) between all pairs of bacterial species for each “Common BE Bacterial Genus.” The Dmean was used to describe the genetic diversity among species within a genus. The genetic distance between a pair of bacteria was calculated with the K80 model using the ‘dist.dna’ function of the ‘ape’ package of R (https://cran.r-project.org/web/packages/ape). We used a nucleotide sequence alignment of the 16S rRNA genes in ‘The All-Species Living Tree’ Project (https://www.arb-silva.de/projects/living-tree/). LTP datasets based on SILVA release 128 were downloaded from Archive (https://www.arb-silva.de/no_cache/download/archive/living_tree/LTP_release_128/). Bacterial genera for which 3 or more taxa (*N* > 2) were available at LTP_release_128 were included in the 16S rRNA diversity analysis. **Table S8.** Genome information. Genome features reported include size (Mb), GC content (%), GCSI (GC skew index), and S value (strength of selected codon usage). A genus was deemed BE if observed in at least 6 publications out of 54. The column “BE” shows the number of references that identified the genera. **Table S9.** Robustness of the study. The genome data set used in this study was tested over two levels: 1) different subsets of bacteria (e.g., Phyla of Proteobacteria, Firmicutes, and Actinobacteria) and also randomly selecting one representative for species that have multiple strains sequenced, and 2) testing different numbers of publications (*n* = 1, 2, 3, 4, 5, and 6) to select for BE genera. **Table S10.** Genomic feature statistical analysis for each MetaMetaDB selected environmental category. Each genomic feature per MetaMetaDB environmental category was analyzed to determine statistical significance between the “Common BE genomes” associated with an environment and the “Common BE genomes” not associated. Significance is indicated by q-value < 0.05 and large effect size by Cliff’s delta |d| > 0.474. (XLSX 3660 kb)

Additional file 2: **Figure S1.** Map of publications used in this study. The 54 publications used in this study are mapped by the closest Köppen latitude and longitude values in order to assign Köppen climate IDs by color (Table S4) (Shades of purple = Dry; Shades of green = Tropical; Shades of grey = Snow; Shades of red/orange = mild temperate). The size of the circle indicates the number of common BE bacterial genera (*n* = 28) identified in the publication. Publications not plotted on the map are those from the International Space Station. **Figure S2.** Descriptive statistics of diversity indices (N, Dmean, PD). Plots of diversity levels between taxa within each genus based on 16S rRNA gene sequences, with scatter plots below the diagonal, histograms on the diagonal, and the Pearson correlation coefficient (Corr) above the diagonal. The diversity levels for each genus were represented by three indices: the number of taxa (N), mean distance (Dmean) between all pairs of taxa, and phylogenetic diversity (PD). **Figure S3.** GC skew plots for *Clostridium perfringens* strain 13 (A) and *Methylobacterium* sp. 4–46 (B). G-language Genome Analysis Environment version 1.9.1 (http://www.g-language.org) was used to generate the GC skew plot. **Figure S4.** Genome size (Mb) distribution among MetaMetaDB selected environmental categories. A boxplot showing the distribution of genome sizes within each “Common BE genus” associated with an environment (purple) compared to the “Common BE genera” not associated (red). **Figure S5.** GC content (%) distribution among MetaMetaDB selected environmental categories. A boxplot showing the distribution of GC content within each “Common BE genus” associated with an environment (purple) compared to the “Common BE genera” not associated (red). **Figure S6.** GCSI distribution among MetaMetaDB selected environmental categories. A boxplot showing the distribution of GCSI within each “Common BE genus” associated with an environment (purple) compared to the “Common BE genera” not associated (red). **Figure S7.** S value distribution among MetaMetaDB selected environmental categories. A boxplot showing the distribution of S value within each “Common BE genus” associated with an environment (purple) compared to the “Common BE genera” not associated (red). **Figure S8.** GC skew plots for *Mycobacterium avium* subsp. *paratuberculosis* K-10 (A) and *Mycobacterium leprae* Br4923 (B). G-language Genome Analysis Environment version 1.9.1 (http://www.g-language.org) was used to generate the GC skew plot. (PDF 7950 kb)

## Abbreviations

BE: Built environment
GC: Guanine and cytosine
GCSI: GC skew index
S value: Strength of selected codon usage bias
Dmean: Mean distance between all pairs of bacteria as a diversity index
PD: Phylogenetic diversity
