# Supplementary material for: Comparative genomics of Bacteria commonly identified in the built environment
Source: BMC Genomics. 2019 Jan 28;20:92. doi: 10.1186/s12864-018-5389-z (PMC6350394; doi:10.1186/s12864-018-5389-z)
Supplement: Supplementary file 2 — Figure S1. Map of publications used in this study. The 54 publications used in this study are mapped by the closest Köppen latitude and longitude values in order to assign Köppen climate IDs by color (Table S4) (Shades of purple = Dry; Shades of green = Tropical; Shades of grey = Snow; Shades of red/orange = mild temperate). The size of the circle indicates the number of common BE bacterial genera (n = 28) identified in the publication. Publications not plotted on the map are those from the International Space Station. Figure S2. Descriptive statistics of diversity indices (N, Dmean, PD). Plots of diversity levels between taxa within each genus based on 16S rRNA gene sequences, with scatter plots below the diagonal, histograms on the diagonal, and the Pearson correlation coefficient (Corr) above the diagonal. The diversity levels for each genus were represented by three indices: the number of taxa (N), mean distance (Dmean) between all pairs of taxa, and phylogenetic diversity (PD). Figure S3. GC skew plots for Clostridium perfringens strain 13 (A) and Methylobacterium sp. 4–46 (B). G-language Genome Analysis Environment version 1.9.1 (http://www.g-language.org) was used to generate the GC skew plot. Figure S4. Genome size (Mb) distribution among MetaMetaDB selected environmental categories. A boxplot showing the distribution of genome sizes within each “Common BE genus” associated with an environment (purple) compared to the “Common BE genera” not associated (red). Figure S5. GC content (%) distribution among MetaMetaDB selected environmental categories. A boxplot showing the distribution of GC content within each “Common BE genus” associated with an environment (purple) compared to the “Common BE genera” not associated (red). Figure S6. GCSI distribution among MetaMetaDB selected environmental categories. A boxplot showing the distribution of GCSI within each “Common BE genus” associated with an environment (purple) compared to the “Common BE genera” not associate [file 12864_2018_5389_MOESM2_ESM.pdf]

Supplementary Information

Comparative genomics of Bacteria commonly identified in the built environment

Nancy Merino[1,2], Shu Zhang[3], Masaru Tomita[4,5] and Haruo Suzuki[4,5*]

1. *Earth-Life Science Institute, Tokyo Institute of Technology, Ookayama, Meguro-ku, Tokyo 152-8550, Japan.*
2. *Department of Earth Sciences, University of Southern California, Stauffer Hall of Science, Los Angeles, CA 90089, USA.*
3. *Global Research Center for Environment and Energy based on Nanomaterials Science, National Institute for Material Science, 1-1 Namiki, Tsukuba, Ibaraki 305-0044, Japan.*
4. *Faculty of Environment and Information Studies, Keio University, Fujisawa, Kanagawa, 252-0882, Japan*
5. *Institute for Advanced Biosciences, Keio University, Tsuruoka, Yamagata, 997-0035, Japan*

Email address:

Nancy Merino: nmerino@elsi.jp

Shu Zhang: zhang.shu@nims.go.jp

Masaru Tomita: mt@sfc.keio.ac.jp

Haruo Suzuki: haruo@sfc.keio.ac.jp

*Corresponding author:

Haruo Suzuki (haruo@sfc.keio.ac.jp)

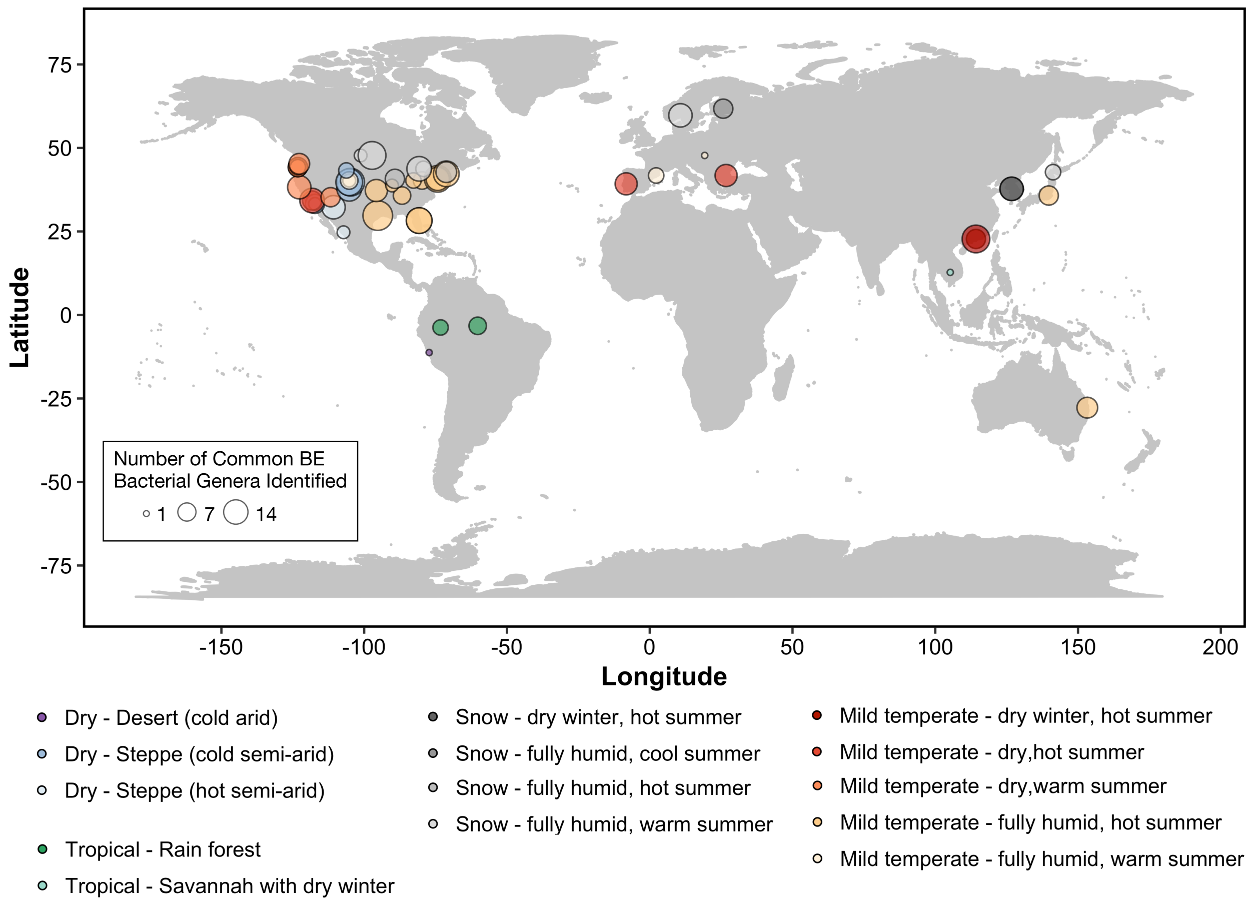

Figure S1. Map of publications used in this study. The 54 publications used in this study are mapped by the closest Köppen latitude and longitude values in order to assign Köppen climate IDs by color (Table S4) (Shades of purple = Dry; Shades of green = Tropical; Shades of grey = Snow; Shades of red/orange = mild temperate). The size of the circle indicates the number of common BE bacterial genera (n = 28) identified in the publication. Publications not plotted on the map are those from the International Space Station.

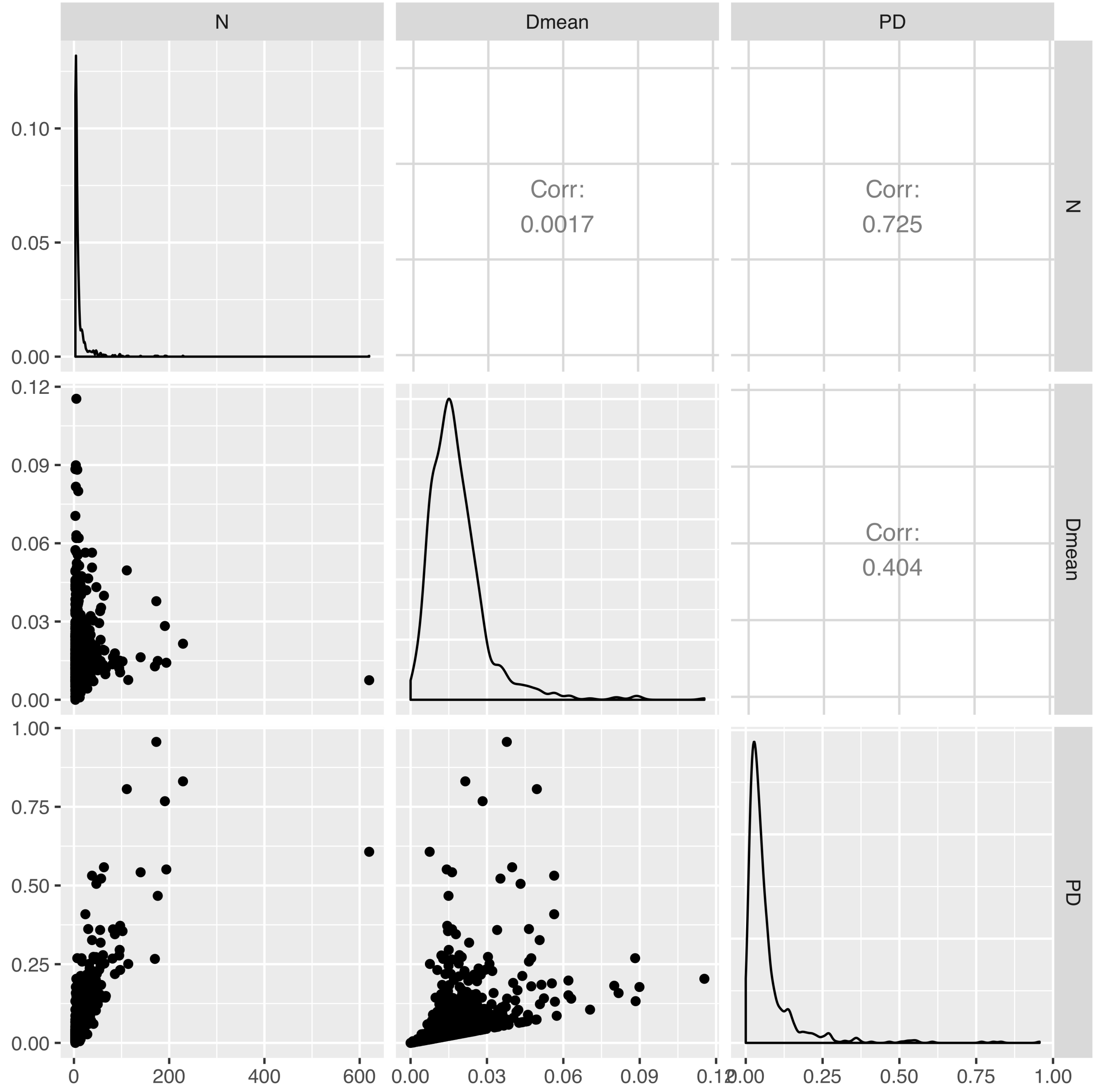

Figure S2. Descriptive statistics of diversity indices (N, Dmean, PD). Plots of diversity levels between taxa within each genus based on 16S rRNA gene sequences, with scatter plots below the diagonal, histograms on the diagonal, and the Pearson correlation coefficient (Corr) above the diagonal. The diversity levels for each genus were represented by three indices: the number of taxa (N), mean distance (Dmean) between all pairs of taxa, and phylogenetic diversity (PD).

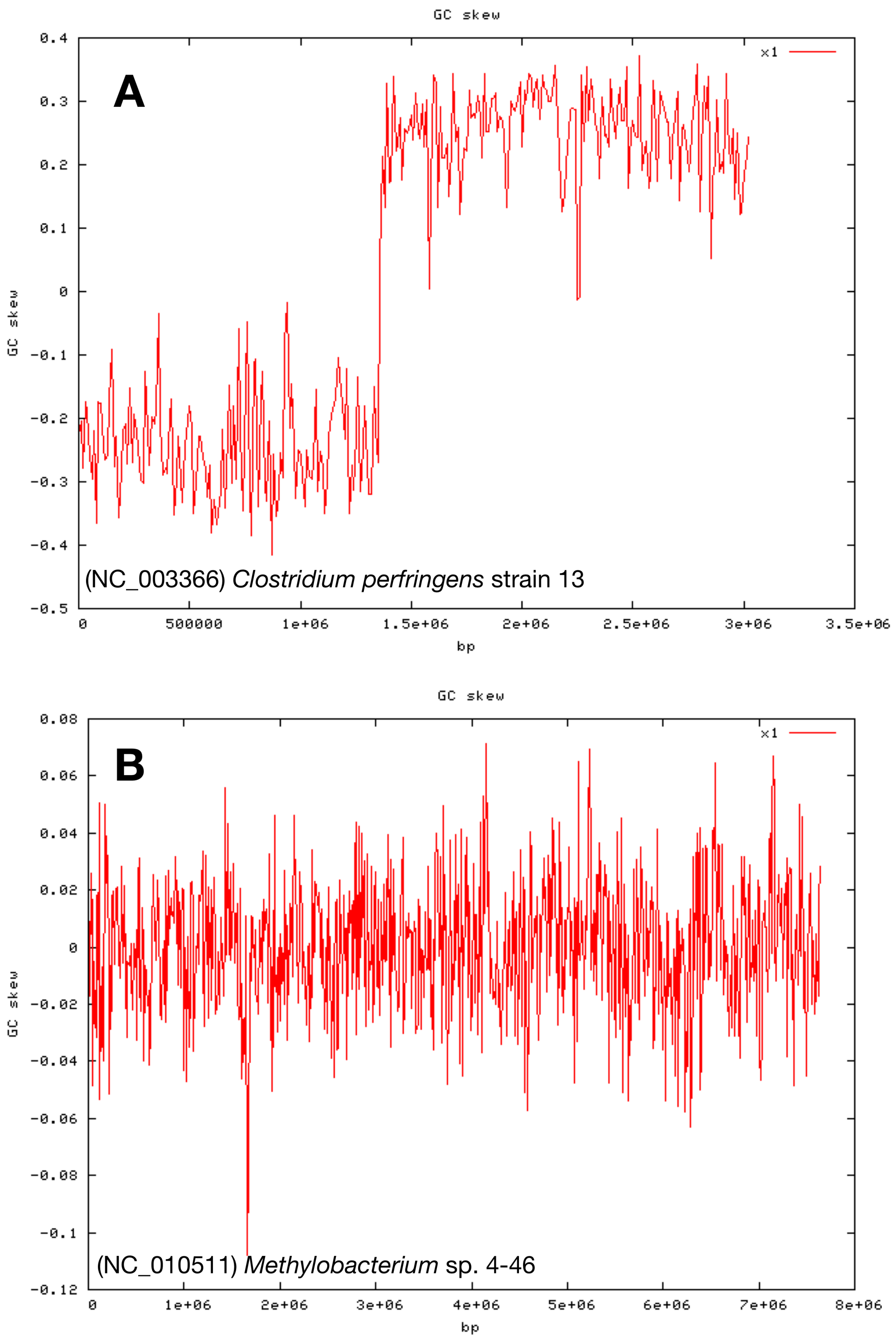

Figure S3. GC skew plots for *Clostridium perfringens* strain 13 (A) and *Methylobacterium* sp. 4-46 (B). G-language Genome Analysis Environment version 1.9.1 (http://www.g-language.org) was used to generate the GC skew plot.

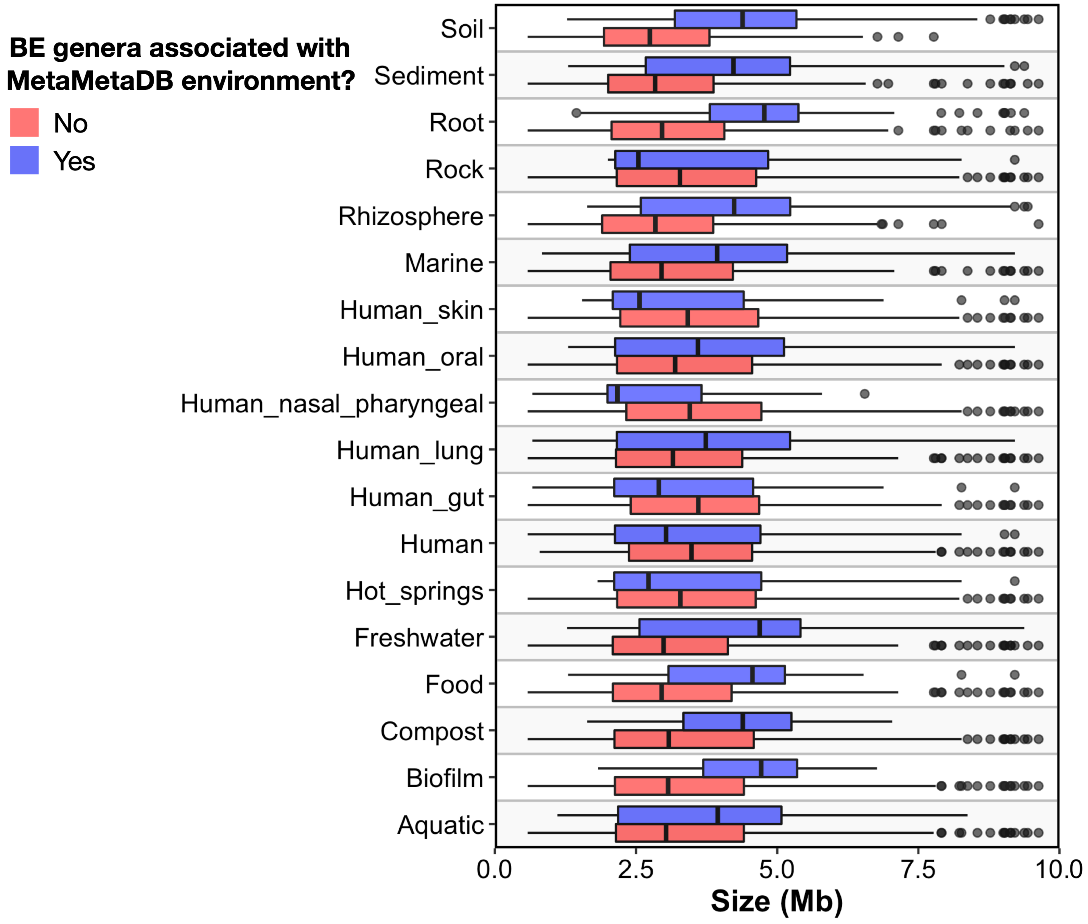

Figure S4. Genome size (Mb) distribution among MetaMetaDB selected environmental categories. A boxplot showing the distribution of genome sizes within each "Common BE genus" associated with an environment (purple) compared to the "Common BE genera" not associated (red).

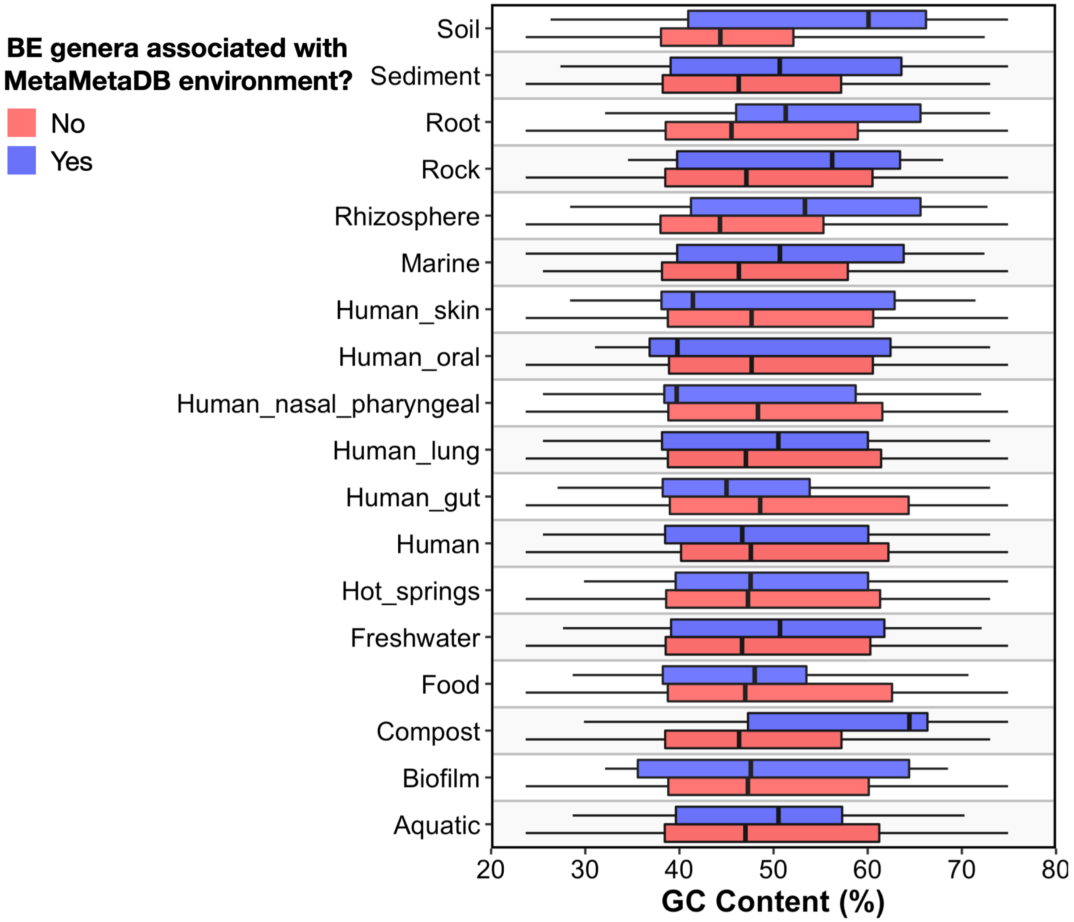

Figure S5. GC content (%) distribution among MetaMetaDB selected environmental categories. A boxplot showing the distribution of GC content within each "Common BE genus" associated with an environment (purple) compared to the "Common BE genera" not associated (red).

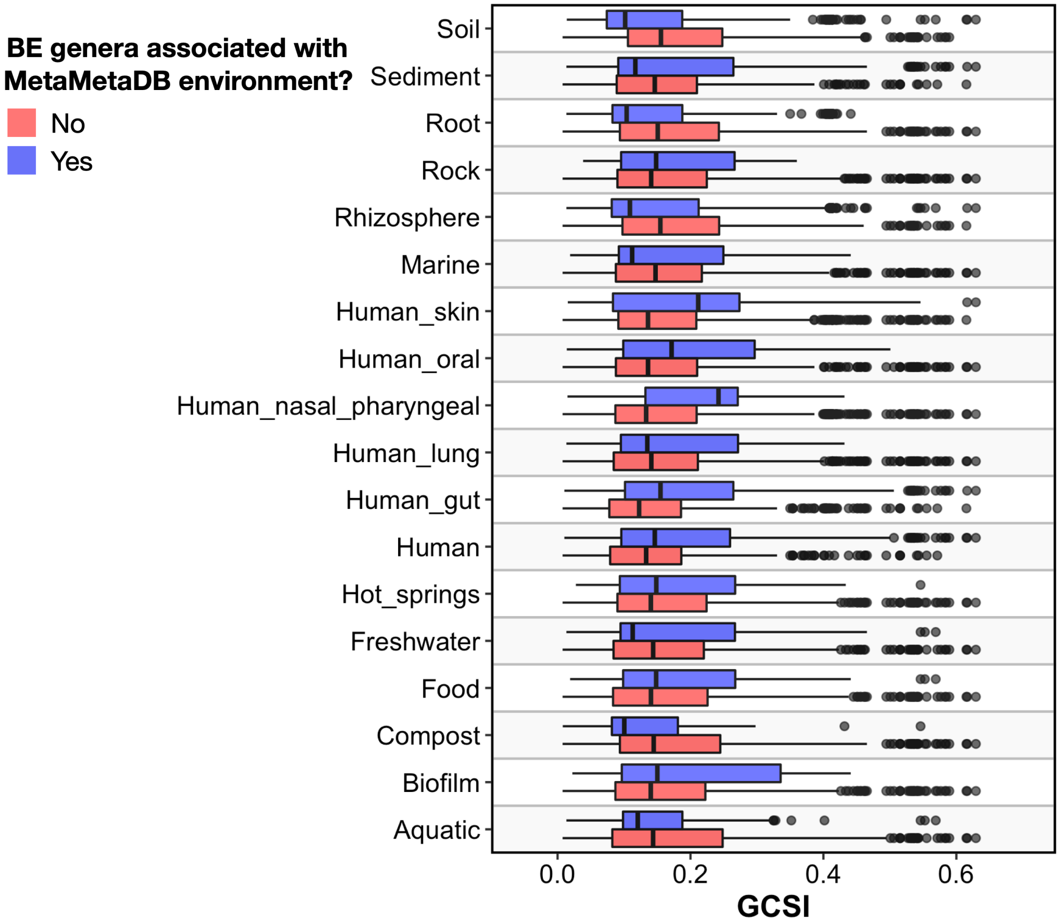

Figure S6. GCSI distribution among MetaMetaDB selected environmental categories. A boxplot showing the distribution of GCSI within each "Common BE genus" associated with an environment (purple) compared to the "Common BE genera" not associated (red).

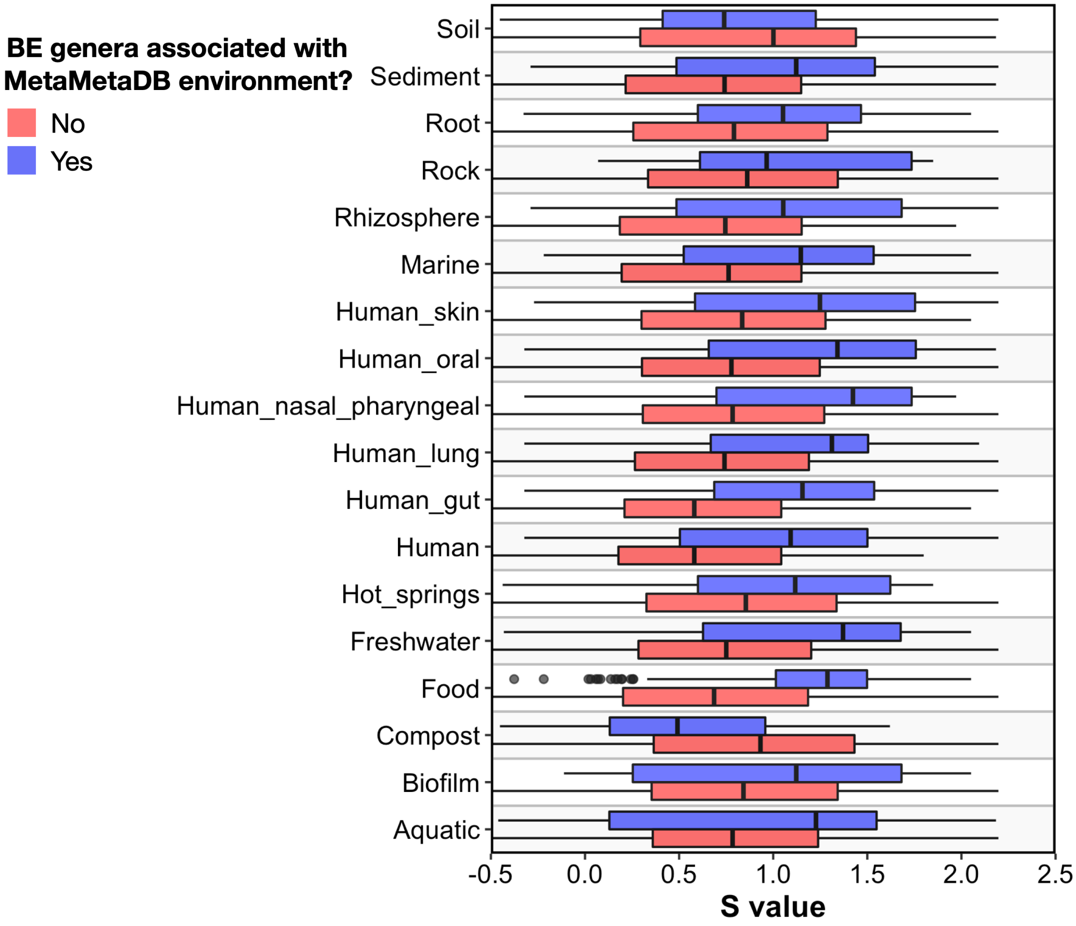

Figure S7. S value distribution among MetaMetaDB selected environmental categories. A boxplot showing the distribution of S value within each "Common BE genus" associated with an environment (purple) compared to the "Common BE genera" not associated (red).

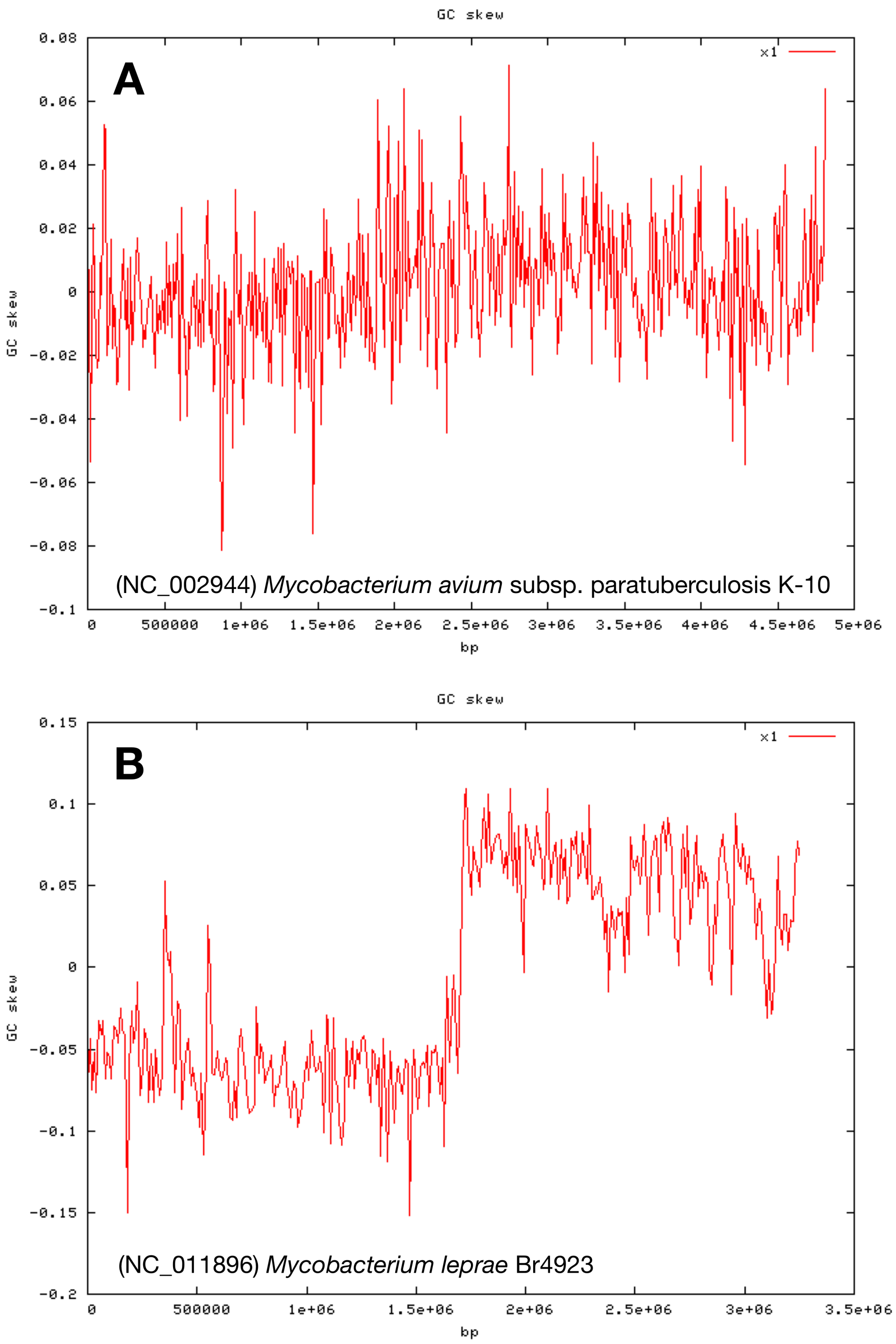

Figure S8. GC skew plots for *Mycobacterium avium* subsp. *paratuberculosis* K-10 (A) and *Mycobacterium leprae* Br4923 (B). G-language Genome Analysis Environment version 1.9.1 (http://www.g-language.org) was used to generate the GC skew plot.
